# Vitamin D Deficiency—A Public Health Issue in Children

**DOI:** 10.3390/children11091061

**Published:** 2024-08-30

**Authors:** Alexandru Herdea, Harun Marie, Adelina Ionescu, Diana-Mihaela Sandu, Sara-Teodora Pribeagu, Alexandru Ulici

**Affiliations:** 1 11th Department of Pediatric Orthopedics, “Carol Davila” University of Medicine and Pharmacy, Bd. Eroii Sanitari Nr. 8, 050474 Bucharest, Romania; alexandru.herdea@umfcd.ro (A.H.); alexandru.ulici@umfcd.ro (A.U.); 2 Pediatric Orthopedics Department, “Grigore Alexandrescu” Children’s Emergency Hospital, 011743 Bucharest, Romania; adelina_ionescu@spitalulgrigorealexandrescu.ro (A.I.);

**Keywords:** 25(OH)D, vitamin D, pediatric, daylight, sunlight exposure, seasonal variation

## Abstract

Background: 25-hydroxyvitamin D [25(OH)D] deficiency is a global health concern, particularly in pediatric populations, with implications for musculoskeletal health and overall well-being. This study aimed to evaluate serum 25(OH)D levels in a pediatric population and assess the prevalence of deficiency and insufficiency. Methods: A retrospective analysis was conducted on data from 741 pediatric patients (2–17 years old) who visited an urban hospital for children for acute respiratory and gastrointestinal conditions in the span of 2 years. Serum 25(OH)D levels were measured using chemiluminescent microparticle immunoassay. Statistical analyses were performed to assess the prevalence of deficiency and insufficiency, seasonal variations, and correlations with age and daylight exposure. Results: Of the 739 pediatric patients analyzed, a substantial proportion exhibited insufficient (31.80%) or deficient (36.54%) serum 25(OH)D levels. While younger age groups generally had higher mean 25(OH)D levels, a negative correlation was observed between 25(OH)D levels and age. Sunlight exposure variations did not significantly impact serum 25(OH)D levels. Despite diverse daylight exposure patterns, there were no significant differences between longer and shorter daylight periods. Conclusions: This study highlights the high prevalence of 25(OH)D deficiency and insufficiency in the pediatric population, emphasizing the need for public health monitoring and targeted supplementation strategies. Findings underscore the importance of regular consultations with healthcare providers to ensure optimal 25(OH)D levels in children, with potential implications for revising current sufficiency thresholds. Addressing 25(OH)D deficiency is crucial for promoting musculoskeletal health and overall well-being in children.

## 1. Introduction

25(OH)D, a fat-soluble micronutrient, plays a critical role in various physiological functions, particularly in skeletal health. Endogenous synthesis through cutaneous exposure to ultraviolet (UV) radiation constitutes the primary source of 25(OH)D for most individuals, with dietary intake playing a secondary role due to its limited presence in common food sources [1]. Upon UV exposure, 25(OH)D3 is synthesized in the skin and subsequently metabolized to 25(OH)D in the circulation [2]. Renal conversion yields the biologically active form 1,25(OH)D, which exerts regulatory control over parathyroid hormone (PTH), calcium, and phosphorus levels [3].

Establishing normative ranges for circulating 25(OH)D levels in children is imperative for clinical assessment. While consensus regarding optimal thresholds remains elusive, the American Academy of Pediatrics and the American Endocrine Society offer differing perspectives, defining insufficiency as serum levels below 20 ng/mL and 30 ng/mL, respectively [4,5,6]. However, levels below these thresholds are universally recognized as indicative of insufficiency or deficiency [7].

25(OH)D receptors have been identified not only in classic target tissues such as bones and the gastrointestinal tract but also in a wide variety of other tissues, including the brain, cardiomyocytes, vascular smooth muscle cells, endothelial cells, pancreatic beta-cells, skeletal muscle, breast, prostate, colon, macrophages, and skin. These receptors exert several pleiotropic effects, and their expression decreases with age. Activated 25(OH)D is implicated in numerous cellular processes, including cellular growth, proliferation and apoptosis, oxidative stress, membrane transport, matrix homeostasis, cell adhesion, and immune system functions. It may also regulate a wide range of genes crucial for healthy aging [8].

Moreover, a negative correlation was previously reported between 25(OH)D levels and pulse wave analysis results, such as pulse wave velocity and central and peripheral blood pressure variables, in children aged 6–18 years, suggesting a predictive value for future cardiovascular events [9].

Children deficient in 25(OH)D often require supplementation, typically administered orally. Guidelines recommend daily doses ranging from 400 to 2000 IU, with higher doses for specific age groups [10]. Despite seasonal variations in sunlight exposure, studies suggest a limited impact on pediatric 25(OH)D levels, with discrepancies observed in specific geographic regions [11,12,13].

In a study of 8594 subjects aged 0 to 94 years, conducted between January 2010 and March 2017, findings revealed the necessity for national programs of 25(OH)D prophylactic supplementation. This recommendation is particularly crucial given the increased incidence of infectious and immunological diseases, as well as cancer, in the Romanian population [14].

Prolonged deficiency predisposes children to various musculoskeletal disorders, including rickets and osteomalacia, which impede bone growth and mineralization [15,16]. Furthermore, 25(OH)D inadequacy heightens the risk of bone fragility and fractures [17].

In light of these concerns, this study aims to retrospectively assess 25(OH)D in pediatric populations, providing insights into prevalence, sunlight exposure variations, and associated health outcomes.

## 2. Materials and Methods

### 2.1. Study Design

This retrospective study aimed to review the records of pediatric patients who attended the pediatric ward for acute symptoms at “Grigore Alexandrescu” Children’s Emergency Hospital, an urban hospital in Bucharest, focusing on the period between September 2021 and September 2023. The ethics committee of “Grigore Alexandrescu” Children’s Emergency Hospital approved this study on 20 September 2023, with the identification number 32.

### 2.2. Participants

Data were extracted from the electronic records stored in the internal system of “Grigore Alexandrescu” Children’s Emergency Hospital. The study analyzed 741 pediatric patients, aged between 2 and 17 years of either gender, who were admitted to the pediatric ward for acute respiratory and gastrointestinal conditions, such as colds or vomiting, and were otherwise healthy children. As part of our routine, every child who undergoes a full blood sample analysis also has their 25(OH)D levels measured.

Demographic data such as age, gender, and location were extracted. Final diagnosis, date of blood sample, clinical and paraclinical exams, and patient histories were also used in this study.

Inclusion criteria included patients with ages between 2 and 17 years, acute symptomatology, 25(OH)D blood samples at least once during the period of the study, complete clinical and paraclinical examinations, a follow-up for at least 12 months, a normal BMI-for-age percentile based on CDC growth charts, with no history of fractures or bone fragility, and, based on parental responses, no recent intake of 25(OH)D supplements in the last 6 months.

The exclusion criteria were as follows: age less than 2 years, age above 18 years, follow-up for less than 12 months, lack of blood samples, lack of complete clinical and paraclinical examinations, chronic gastrointestinal disease, chronic kidney disease, history of cystic fibrosis, Crohn’s disease or celiac disease, asthma, bone metabolic disorders, tumors/cancer, cardiovascular diseases, diabetes, osteoporosis, rickets or sarcopenia, lack of patient history, children with low BMI or malnutrition, patients with obesity, history of fractures.

Exclusion criteria were important for distinguishing between otherwise healthy children and chronic diseases that interfere with 25(OH)D metabolism. Patients with records of fractures were also excluded due to a possible link with 25(OH)D deficiency.

A follow-up period of at least 12 months was used to exclude patients who, despite presenting with acute symptoms, had an underlying chronic pathology. After discharge, patients were assessed in an outpatient clinic at intervals of 7 days, 21 days, 3 months, 6 months, and 12 months.

### 2.3. Biochemical Analysis

Blood samples were obtained from patients who visited the pediatric ward in the conditions mentioned above. Levels of serum 25(OH)D were determined using the Architect i2000 (II) chemiluminescent microparticle immunoassay (CMIA) manufactured by Abbott Laboratories, Abbott Park, IL, USA. The assay’s precision and accuracy were validated according to the manufacturer’s protocols, and it has been compared to standardized 25(OH)D measurements as detailed in Avici et al. [18].

### 2.4. Outcome Measures

The aim of this study was to assess the prevalence of 25(OH)D deficiency amongst the pediatric population. In addition, this study also followed secondary outcomes such as the relationship between serum 25(OH)D levels and daylight exposure variations, its relationship with the patients age, and the cumulative frequency distribution of serum 25(OH)D levels amid the pediatric population visiting the hospital. The Endocrine Society’s Clinical Practice Guidelines suggest that a 25(OH)D level of 30 ng/mL is adequate for both children and adults, with levels below 20 ng/mL (50 nmol/L) classified as deficient and those between 21 and 29 ng/mL (55–75 nmol/L) considered insufficient [6].

The study population was categorized based on the duration of daylight exposure at the time of blood sample collection. Patients whose blood samples were collected during the months classified as having longer daylight exposure (April to August) were assigned to the longer daylight exposure group. Conversely, patients whose blood samples were collected during the months classified as having shorter daylight exposure (October to February) were assigned to the shorter daylight exposure group. Patients whose blood samples were collected during March or September were placed into two separate groups in order to analyze the impact of these transitional months, which follow the colder and hotter periods, respectively. Additionally, since daylight and nighttime are equal in March and September in Romania, these months provide a unique opportunity to study the effects of equal daylight exposure on serum 25(OH)D levels. Thus, 4 distinct groups were created. This categorization allowed for an analysis of seasonal variations in serum 25(OH)D levels, taking into consideration the significance of sunlight exposure on 25(OH)D synthesis.

### 2.5. Statistical Analysis

Patient data was collected and stored electronically from the hospital’s medical records system. This data included both categorical qualitative data and quantitative information. Categorical qualitative data consisted of gender and the date of blood sample collection. Quantitative data included the patients’ age at diagnosis and their 25(OH)D levels at that time. Descriptive statistics were applied to the data, utilizing measures like frequencies, sex incidence, odds ratios, chi-square tests, and *p*-values. Statistical significance was determined by a *p*-value of less than 0.05, with a 95% confidence interval used throughout the analysis. The statistical analyses were conducted using IBM SPSS Statistics (Version 26) and Microsoft Excel Office 2016 (Microsoft, Redmond, WA, USA).

## 3. Results

Table 1 displays the distribution of gender across various age groups and across the three serum 25(OH)D intervals. Each age group is delineated by a range of years, with corresponding counts for females and males, along with the total number of individuals in each age group. The total counts reflect the cumulative sum of females, males, and individuals across all age groups, amounting to 372 females (50.2%), 369 males (48.9%), and a total of 741 individuals, respectively. 234 (31.6%) individuals had normal 25(OH)D values, 235 (31.8%) had insufficient values, and 270 (36.5%) were deficient in 25(OH)D. Also, 177 children (23.9%) came from rural areas, while 564 patients (76.1%) were from urban areas.

From 741 patients, we could analyze only 739 because two patients had improper blood samples. The mean 25(OH)D level of the 739 patients analyzed is 27.0 ng/mL, which indicates a general insufficiency of serum 25(OH)D within the observed population. The relatively high standard deviation (14.2 ng/mL) indicates considerable variability in 25(OH)D levels among individuals. The wide range between the minimum and maximum values (5.1 ng/mL to 126 ng/mL) underscores the diversity of 25(OH)D levels within the population, with some individuals exhibiting levels considered insufficient or excessive.

The mean 25(OH)D level for females is 27.5 ng/mL, while for males it is slightly lower at 26.5 ng/mL. Overall, the mean 25(OH)D level for the entire observed population is 27.0 ng/mL. While the mean 25(OH)D level is slightly higher for females compared to males, the difference is not substantial (*t*-test, *p* > 0.05). While the analysis reveals comparable mean 25(OH)D levels between genders, with females showing a slightly higher mean level, they also show a slightly higher variability. No significant difference was found between rural or urban areas (*p* > 0.05, *t*-test).

The present paper observed serum 25(OH)D levels categorized into different age groups: 2–3 years, 4–6 years, 7–10 years, 11–14 years, and 15–17 years. The mean 25(OH)D levels vary across different age groups, ranging from 20.3 ng/mL to 39.3 ng/mL. 25(OH)D levels exhibit variability across different age groups, with younger age groups generally showing higher mean levels compared to older age groups.

The Pearson correlation coefficient between 25(OH)D levels and age is approximately −0.91. This reflects a strong negative correlation, indicating that as age increases, 25(OH)D levels tend to decrease. The correlation is statistically significant at the 0.01 level (two-tailed), with an approximate *p*-value of 0.02, suggesting that this observed relationship is unlikely to have occurred by chance.

It can be observed that in the 7–17-year-old age group, 25(OH)D value decreases, presenting the lowest value in the 15–17-year-old age group. The mean 25(OH)D level for the 11–14 year age group is approximately 22.3 ng/mL, while for the 15–17 year age group, it is about 21.3 ng/mL. This suggests that, on average, the 25(OH)D levels are slightly higher in the 11–14 year age group compared to the 15–17 year age group, but the difference is relatively small. It can be concluded that in the last three groups, mean 25(OH)D levels are insufficient (*p* < 0.05).

Table 2 provides insight into the distribution of 25(OH)D according to daylight exposure. Romania and Eastern Europe have longer daylight between the months of April–August and shorter daylight between the months of October and February, whereas in March and September, daytime is considered to be equal to nighttime [19]. However, there was no statistically significant difference in mean 25(OH)D levels between patients from the periods of daylight exposure (*p* > 0.05).

Age groups did not show significant variations of mean serum 25(OH)D levels. Only two of the age groups (0–3 years) and (4–6 years) displayed sufficient mean concentrations of 25(OH)D. The other age groups expressed insufficient mean concentration levels of 25(OH)D.

## 4. Discussion

Our study presents a novel finding that 25(OH)D levels in adolescents do not significantly vary across different periods of daylight exposure. This is particularly noteworthy given the widespread assumption that 25(OH)D levels are closely tied to seasonal changes, with the expectation of lower levels during periods of shorter daylight. This finding challenges the existing paradigm and suggests that factors other than daylight exposure may play a more critical role in maintaining 25(OH)D levels in this age group.

This study seeks to provide insight into the relationship between daylight exposure due to year-round variation and serum levels of 25(OH)D in the pediatric population who visited our hospital. We decided, in our aim to obtain the most objective results, to split the assessed pediatric population into four groups according to daylight exposure variation throughout the year at the time of blood collection to compare the concentration of the circulating 25(OH)D. Our study demonstrates that 25(OH)D levels did not show significant seasonal variation among the different periods of daylight exposure. This finding is particularly important as it challenges the commonly held notion that 25(OH)D levels fluctuate significantly with changes in daylight exposure in adolescents.

Daylight exposure in Romania and Eastern European countries varies significantly due to geographical location and seasonal changes. In Romania, as well as other Eastern European countries, daylight saving time (DST) is observed, which impacts the amount of daylight the population is exposed to during different times of the year [20].

In Romania, DST starts on the last Sunday of March and ends on the last Sunday of October, which means clocks are set forward by one hour in March and set back by one hour in October. This adjustment aims to make better use of daylight during the longer days of summer [21,22]. The extended daylight hours during DST can increase the population’s exposure to sunlight, which is beneficial for 25(OH)D synthesis.

The amount of daylight or sunshine varies significantly with the seasons. During the summer, days are longer, providing more hours of sunlight. Conversely, in winter, daylight hours are shorter, especially in higher latitudes like those of Romania and Eastern Europe. This seasonal variation plays a crucial role in 25(OH)D levels among the population, as longer exposure to sunlight in summer helps boost 25(OH)D levels, while shorter days in winter might lead to deficiencies [21,23].

For example, during the summer solstice, Bucharest experiences approximately 15.5 h of daylight, whereas during the winter solstice, it experiences only about 8.5 h. This substantial difference underscores the importance of considering seasonal changes when discussing sunlight exposure and 25(OH)D levels in these regions [22]. These variations highlight the need for 25(OH)D supplementation, especially during the winter months when natural sunlight exposure is insufficient. Additionally, fortification of foods with 25(OH)D can be an effective strategy to maintain adequate 25(OH)D levels year-round.

Reduced levels of serum 25(OH)D are proving to be a significant public health issue globally with an extensive reach, encompassing all age groups. However, the extent of its prevalence varies considerably among different nations and demographic subsets due to a multitude of influencing factors. These factors include variations in skin pigmentation, clothing, sun exposure patterns, season, individual lifestyles, and environmental nuances. Obesity, malabsorption disorders, kidney or liver disorders, certain malignancies, and genetic predispositions further contribute to the challenge of 25(OH)D inadequacy. Moreover, prenatal influences like maternal 25(OH)D deficiency and premature birth, alongside postnatal elements such as prolonged breastfeeding, insufficient dietary intake, darker skin tones, or limited sun exposure, contribute to this diverse list [24]. Certain medications, including antiepileptic drugs, glucocorticoids, anti-estrogens, and antiretroviral agents, can affect serum levels and the metabolism of 25(OH)D, necessitating additional monitoring and care for patients undergoing such treatments. To ensure an adequate response to bisphosphonate therapy in children with severe osteogenesis imperfecta, maintaining proper 25(OH)D levels is crucial. Preliminary research also suggests that 25(OH)D may influence the lipid-lowering effects of statins (HMG-CoA reductase inhibitors) and the antibacterial efficacy of antitubercular medications, identifying these as supplementary risk factors [25].

Several studies have documented the prevalence and risk factors associated with low 25(OH)D levels among European adolescents, noting regional and seasonal variations. The OPTImal FORification of 25(OH)D (OPTIFORD) study [26,27], measured 25(OH)D levels in adolescent girls (average age 12.5 years) from Denmark, Finland, Poland, and Ireland (latitudes between 51° and 60° N) between February and March. The prevalence of 25(OH)D deficiency (defined as <7.86 ng/mL) varied from 26% to 51%, with over 90% of the adolescents exhibiting suboptimal 25(OH)D levels (<15.72 ng/mL). High winter prevalence of deficiency has also been observed in Southern Europe; for instance, 47% of Greek adolescents had serum 25(OH)D levels below 7.86 ng/mL [28,29].

In a study made between 2012 and 2014, 527 Danish children who had their blood drawn twice (in the spring and the fall) revealed that just 0.4% and 0.8% of the children were 25(OH)D deficient in the fall, compared to 18% of boys and 9% of girls in the spring. Regarding 25(OH)D deficiency, this was observed in 42% of boys in the spring (which decreased to 16% in the autumn); a higher percentage of girls (50%) had 25(OH)D deficiency in the spring (which decreased to 12% in the fall) [30].

A systematic review of 25(OH)D levels in Southern European countries found an unexpectedly high prevalence of low 25(OH)D status in Southern Europe and the Eastern Mediterranean regions, despite the ample sunshine in these areas [31].

In our study, we found no statistically significant correlation between the serum levels of 25(OH)D and the influence of the number of hours of daylight exposure. Romania is characterized by a diverse climate, experiencing all four distinct seasons: spring, summer, autumn, and winter. This seasonal variation brings about a wide range of ambient temperatures throughout the year, making it a dynamic and diverse environment for its residents. Additionally, Romania’s geography offers diverse landscapes, from mountainous regions in the north to plains and beaches along the Black Sea coast.

The mean of the serum concentrations of 25(OH)D throughout the duration of the study for each gender falls within the limits of the insufficient range with females showing slightly higher mean than men. Mean serum 25(OH)D during longer and shorter daylight periods also fell within the insufficient range; the mean value in March, when daylight and nighttime is equal also fell in the insufficient range value, whereas mean value of serum 25(OH)D in September was the only sufficient value. Differences between age groups were found, with only (2–3 years) and (4–6 years) groups displaying sufficient levels when compared to other age groups; however, the mean concentrations of serum 25(OH)D for both age groups were slightly above the lower limit of the sufficient interval, as seen in Table 3. Our findings, based on the negative correlation coefficient with statistical significance, suggest that there is a tendency for lower 25(OH)D levels as age increases; this further aids the argument that 25(OH)D supplementation, especially as children age is a necessity and should be implemented on national levels, tailored specifically to different population subgroups, with emphasis on the higher risk groups.

Compared to a study conducted to evaluate the 25(OH)D status in 510 healthy children aged 4–15 living in Jeddah, Saudi Arabia, whose population with its naturally darker skin pigmentation experiences extensive sun exposure due to the country’s desert climate, characterized by abundant sunshine year-round, that found (13.72%) had normal 25(OH)D levels ranging from 20 to 70 ng/mL, 58.82% had relative 25(OH)D deficiency, and 27.45% had severe deficiency [32]. However, cultural practices, such as wearing traditional clothing that covers much of the body, and religious practices, such as fasting during Ramadan, may influence the extent of sun exposure and dietary intake for individuals. Our findings show that of the 739 healthy Caucasian children 234 (31.66%) patients had a normal 25(OH)D level, 235 (31.80%) had a 25(OH)D insufficiency, and 270 (36.54%) had deficient 25(OH)D levels.

A study that followed the impact of using melatonin, calcium, and 25(OH)D on patients with idiopathic scoliosis found that 25(OH)D deficiency may be linked to scoliosis progression; suggesting that supplementation with 25(OH)D, particularly for patients with low serum levels, could be a valuable new approach in treating this condition [33].

The duration of sunlight exposure throughout the year had no significant effects on serum levels of 25(OH)D. Patients whose blood samples were collected in the longer daylight periods had no statistically significant difference in serum 25(OH)D levels compared to the ones whose blood samples were collected in the shorter daylight period or transitional months (*p* > 0.005). A 2024 study assessing serum 25(OH)D levels in 2317 Italian children and adolescents under 18 years old from Northern Sardinia found that their vitamin D levels were not significantly impacted by the pandemic’s restrictive measures. The study revealed that 25(OH)D concentrations generally stayed within the sufficient range throughout the pandemic and showed no substantial difference compared to the pre-pandemic levels [34]. This further adds to the findings in our study: sunlight exposure did not significantly influence serum 25(OH)D levels; our study reinforces existing evidence that sunlight exposure alone may not be sufficient to significantly raise serum 25(OH)D levels or address deficiency. Despite a desirable serum 25(OH)D level of 20 ng/mL, in the Middle East and North Africa, 25(OH)D deficiency is widespread, with prevalence rates ranging from 30% to 90%, concluding that Rickets and osteomalacia still occur in these sunny regions [35].

Adequate 25(OH)D levels are crucial for efficient calcium absorption in the intestines and for the development and maintenance of healthy bones. An observational study that followed if low 25(OH)D levels have a role in pediatric morbidity and mortality after 52 postmortem examinations reached the conclusion that 25(OH)D deficiency (the most common form of pediatric metabolic bone disease) is preventable and treatable. Profound hypocalcemia due to severe 25(OH)D deficiency can cause unexpected death in babies and young children [36]. While bone diseases associated with 25(OH)D deficiency, such as rickets and osteomalacia, are rare in Western populations, they remain prevalent in regions like Asia, Africa, and the Middle East [37,38]. Dietary calcium deficiency and 25(OH)D deficiency represent the two extremes in the pathogenesis of nutritional rickets, with a combination of both conditions falling in between [39].

Even though Africa and parts of Asia experience a sunny climate with higher UV radiation levels [40], according to a review by Mithal et al., hypovitaminosis (serum 25(OH)D < 30 ng/mL) is prevalent worldwide, with levels below 10 ng/mL (25 nmol/L) being most common in South Asia and the Middle East. The primary factors significantly associated with lower 25(OH)D levels include older age, female sex, higher latitude, winter season, darker skin pigmentation, reduced sunlight exposure, dietary habits, and the lack of 25(OH)D fortification in foods [41].

The impact of early nutrition is crucial for future health and nutritional status. The study on feeding practices among Romanian children in the first year of life highlights significant socio-economic influences on complementary feeding. It was found that children from rural areas, low-income families, and those with less educated mothers are at a higher risk for inappropriate feeding practices. These factors could potentially contribute to the observed variations in 25(OH)D levels and other health outcomes in our study. Addressing these disparities through targeted educational programs and material support could improve health outcomes, including those related to 25(OH)D levels [42].

Dietary sources of 25(OH)D, such as meat and fish, contribute to 25(OH)D levels, as highlighted in the EPIC-Oxford study [43]. The influence of dietary intake and supplementation should be considered when interpreting 25(OH)D status.

Food fortification with 25(OH)D could play a crucial role in combating widespread 25(OH)D deficiency, particularly in regions with limited sunlight exposure. Fortifying staples such as milk, cereals, and juices have been shown to effectively increase 25(OH)D levels in the population. For instance, studies indicate that fortified foods can significantly improve serum 25(OH)D concentrations, providing a practical solution for those unable to achieve sufficient levels through diet or sunlight alone [44]. Additionally, organizations like the World Health Organization and the European Food Safety Authority endorse fortification as a public health measure to address deficiencies in vulnerable groups, including children and the elderly [45,46]. Implementing these measures in Romania and other Eastern European countries could help mitigate the seasonal variations in 25(OH)D synthesis due to limited daylight exposure during the winter months.

It is important to note that 25(OH)D levels can be influenced by acute inflammatory conditions. The study by Oscanoa et al. demonstrated that serum 25-hydroxyvitamin D concentrations are significantly lowered in response to acute inflammatory markers, particularly in patients with SARS-CoV-2 infection. This suggests that measurements taken during periods of acute illness may not accurately reflect normal 25(OH)D levels. However, in this study, patients presented without fever or SARS-CoV-2 infection, which can influence vitamin D levels. While the response time of 25(OH)D levels to inflammatory markers or fever is not specifically addressed in the Oscanoa et al. study, it is generally understood that serum concentrations of vitamin D can be affected by these factors over time. The limitations highlighted by Oscanoa et al. underscore the need for future studies to measure 25(OH)D levels in non-acute settings or account for inflammatory conditions when assessing vitamin D status to ensure more accurate evaluations [47].

Our study’s strengths reside in its stride to offer valuable insight into the relationship between daylight exposure and serum 25(OH)D levels. As study limitations we can list variability of clothing and sunlight exposure patterns, this study may not have captured the full picture of individual sunlight exposure habits, dietary preferences, time spent outdoors or travel history close to the time of blood sample collection. Another limitation of this study is the lack of consideration for air pollution, which is known to be associated with 25(OH)D insufficiency. Air pollution, along with smoking, can reduce the skin’s ability to synthesize 25(OH)D from sunlight, potentially confounding the relationship between daylight exposure and 25(OH)D levels [8]. Additionally, while obesity is also a known factor associated with 25(OH)D insufficiency, it was an exclusion criterion in our study.

Further research efforts should aim for longitudinal data collection, following the same patients over an extended period of time with blood samples being collected at least twice each year, one of which being in the same month each time would provide valuable information about seasonal variations in 25(OH)D levels. Investigating the correlation across different age groups could reveal how 25(OH)D metabolism changes as the pediatric population ages. Future studies could explore the role of dietary intake, physical activity, or genetic factors in maintaining 25(OH)D levels in adolescents, regardless of seasonal changes.

25(OH)D insufficiency/deficiency should be classified as public health concerns as multiple studies have demonstrated their link to various ailments; further, population-wide blood sample collection and serum levels of 25(OH)D should be periodically determined, followed by supplementation tailored to population subgroups and individuals, if needed, keeping higher risk groups under closer supervision.

Prophylactic 25(OH)D supplementation throughout childhood can be beneficial. While dietary sources and sunlight exposure contribute to the 25(OH)D stock in the body, supplementation is often necessary to ensure sufficient serum 25(OH)D (25(OH)D) levels in the pediatric population. We consider that periodically consulting with a healthcare professional is recommended to establish the most appropriate 25(OH)D supplementation regimen for every child, with emphasis on at-risk children.

The current definition of 25(OH)D sufficiency, particularly in the pediatric population, might be worthy of reevaluation due to the alarming rise in 25(OH)D deficiency and insufficiency rates. Currently, a serum 25(OH)D level greater than 20 ng/mL (50 nmol/L) has been considered sufficient. However, this threshold may not adequately promote optimal health outcomes in children. Therefore, reevaluating sufficiency thresholds and adopting a personalized approach with supplementation potentially exceeding current levels for at-risk children, alongside promoting safe sun exposure practices, could be crucial steps towards ensuring optimal 25(OH)D levels and improving the overall health of the pediatric population. Implications for clinical practice include recognizing that adolescents, particularly those aged 15–17 years, may be at risk for 25(OH)D deficiency regardless of seasonal changes. This is critical as low 25(OH)D levels in this age group may predispose them to future cardiovascular risks, given the negative correlation previously reported between 25(OH)D levels and arterial stiffness and blood pressure parameters.

## 5. Conclusions

The high prevalence of 25(OH)D deficiency suggests the importance of public health monitoring and targeted supplementation, with closer attention to high-risk groups.

Regular consultations with healthcare providers can ensure optimal 25(OH)D levels in children through personalized and even prophylactic supplementation plans.

The high prevalence of 25(OH)D deficiency in children suggests revising sufficiency levels; taking into consideration a higher threshold for the sufficiency interval may be beneficial.

## Figures and Tables

**Table 1:** Distribution of gender and 25(OH)D status levels across age groups. N—number of patients.

Age	25(OH)D Mean (ng/mL)						N
	Deficient		Insufficient		Normal		
	M	F	M	F	M	F	
2–3 years	15.6	16.1	26.0	25.2	40.6	49.4	163
4–6 years	14.9	19.1	24.0	24.9	45.2	37.8	168
7–10 years	13.4	14.4	23.6	24.8	39.6	36.9	160
11–14 years	13.8	13.3	24.5	24.3	45.0	39.8	169
15–17 years	13.5	14.1	25.0	24.1	37.0	39.4	81
	14.8		24.0		41.1		
N	270 (36.5%)		235 (31.8%)		234 (31.6%)		

**Table 2:** Daylight exposure variation of 25(OH)D. N—number of patients. SD—standard deviation.

Exposure	N	25(OH)D Mean (ng/mL)	25(OH)D Mean (SD) (ng/mL)
Longer Daylight	327	27.3	13.1
Shorter Daylight	266	26.9	13.5
March	81	26.2	20.4
September	67	30.5	14.6

**Table 3:** Descriptive statistics for 25(OH)D levels by age group. N—number of patients. SD—standard deviation.

Age	25(OH)D Mean (ng/mL)	N	25(OH)D Mean (SD) (ng/mL)	CI 95%
2–3 years	34.8	162	17.7	34.8 ± 2.7 (±7.9%) [32.1–37.6]
4–6 years	30.2	168	14.1	30.2 ± 2.1 (±7.1%) [28.1–32.4]
7–10 years	24.5	160	10.4	24.4 ± 1.6 (±6.6%) [22.8–26.1]
11–14 years	22.3	169	11.5	22.3 ± 1.7 (±7.8%) [20.5–24.0]
15–17 years	21.2	80	9.4	21.2 ± 2 (±9.7%) [19.2–23.3]
Total	27.0	739	14.2	27 ± 1 (±3.8%) [25.9–28]

## Data Availability

The data presented in this study are available on request from the corresponding author. The data are not publicly available due to privacy concerns and ethical considerations.
